# Ultrasound-guided percutaneous tracheostomy in critically ill obese patients

**DOI:** 10.1186/cc11233

**Published:** 2012-03-05

**Authors:** Pierre-Grégoire Guinot, Elie Zogheib, Sandra Petiot, Jean-Pierre Marienne, Anne-Marie Guerin, Pauline Monet, Rody Zaatar, Hervé Dupont

**Affiliations:** 1 Department of Anaesthesiology and Critical Care Medicine, Amiens University Hospital, Place Victor Pauchet, 80054 Amiens, France; 2 Critical Care Department, Beauvais General Hospital, Avenue Leon Blum, 60021 Beauvais, France; 3 Radiology Department, Amiens University Hospital, Place Victor Pauchet, 80054 Amiens, France; 4 Head and Neck Surgery Department, Amiens University Hospital, Place Victor Pauchet, 80054 Amiens, France; 5 INSERM UMR1088, Jules Verne University of Picardy, 12 rue des Louvels, 80000 Amiens, France

## Abstract

**Introduction:**

The purpose of this study was to evaluate the feasibility of ultrasound (US)-guided percutaneous tracheostomy (PCT) and the incidence of complications in critically ill, obese patients.

**Methods:**

Fifty consecutive patients were included in a prospective study in two surgical and critical care medicine departments. Obesity was defined as a body mass index (BMI) of at least 30 kg/m^2^. The feasibility of PCT and the incidence of complications were compared in obese patients (n = 26) and non-obese patients (n = 24). Results are expressed as the median (25^th^-75^th ^percentile) or number (percentage).

**Results:**

The median BMIs were 34 kg/m^2 ^(32-38) in the obese patient group and 25 kg/m^2 ^(24-28) in the non-obese group (p < 0.001). The median times for tracheostomy were 10 min (8-14) in non-obese patients and 9 min (5-10) in obese-patients (p = 0.1). The overall complication rate was similar in obese and non-obese patient groups (35% vs. 33%, p = 0.92). Most complications were minor (hypotension, desaturation, tracheal cuff puncture and minor bleeding), with no differences between obese and non-obese groups. Bronchoscopic inspection revealed two cases of granuloma (8%) in obese patients. One non-obese patient developed a peristomal skin infection, which was treated with intravenous antibiotics. Ultrasound-guided PCT was possible in all enrolled patients and there were no surgical conversions or deaths.

**Conclusions:**

This study demonstrated that US-guided PCT is feasible in obese patients with a low complication rate. Obesity may not constitute a contra-indication for US-guided PCT. A US examination provides information on cervical anatomy and hence modifies and guides choice of the PCT puncture site.

**Trial registration:**

ClinicalTrials.gov: NCT01502657.

## Introduction

The increasing prevalence of obesity worldwide has prompted rapid growth in the proportion of obese patients admitted to intensive care units (ICUs). The management and care of such patients are complex since obesity is associated with a longer duration of mechanical ventilation and a longer stay in the ICU [1,2]. During their stay in the ICU, obese patients may require tracheostomy; this procedure is commonly performed when ventilatory weaning fails or when prolonged mechanical ventilation is needed [3,4]. Since the generalization of percutaneous tracheostomy (PCT), many different types of complication have been described [5]. PCT under bronchoscopic control has been developed in this context. Bronchoscopy uses transillumination to indicate the puncture site, confirm the needle position, and control the dilatation and positioning of the tracheostomy tube [6-8]. However, bronchoscopy does not identify the vascular structures or the thyroid gland in the neck region and thus does not prevent complications linked to local organ lesions (punctured vessels or a punctured thyroid) [9]. This is particularly true in obese patients, in whom anatomical landmarks may not be easily identified in a physical examination. Many authors have reported a higher incidence of complications in obese patients [10,11].

In recent years, technical progress and low invasiveness have meant that ultrasound (US) is increasingly used in clinical practice in anesthesia and critical care [12]. This is notably the case for improving procedures (central venous cannulation, arterial cannulation, and peripheral nerve block) in obese patients [13]. With respect to PCT, several studies have demonstrated the value of US mapping of the neck region prior to PCT [14-16]. Indeed, US examination often modifies the puncture site with respect to that chosen solely on the basis of anatomical palpation data [17]. Recently, Rajajee and colleagues [18] demonstrated the feasibility of US-guided PCT in a neurosurgical ICU. They also reported the safety of US-guided tracheostomy in three obese patients. However, to the best of our knowledge, there are no published data on the US-guided PCT procedure in obese patients.

Hence, the objectives of our study were to evaluate the feasibility of US guidance, describe any difficulties, and evaluate the incidence of complications in an obese population in surgical and medical critical care departments. We prospectively compared this obese population with a non-obese population.

## Materials and methods

This study was a prospective, two-center cohort study of 50 consecutive patients. The protocol was approved by the local independent ethics committee (Comité de Protection des Personnes Nord Ouest, Amiens, France). All patients (or, for unconscious patients, the next of kin) gave their written informed consent to participation. We enrolled all patients who were hospitalized in the ICUs at Amiens University Hospital or Beauvais General Hospital and for whom PCT was indicated. Exclusion criteria were as follows: age under 18 years, coagulation disorders (platelet count of below 80,000 mm^-3 ^and an international normalized ratio of at least 1.2), infection at the puncture site, and emergency tracheostomy.

### The percutaneous tracheostomy technique

PCT was performed by using the single-step, progressive Ciaglia Blue Rhino technique, as described previously [18,19]. The PCT set consisted of a puncture needle, a guide wire, a small dilator, a curved dilator with a hydrophilic coating, and a tracheostomy tube (Tracoe^® ^experc dilatation set; Pouret Medical, Clichy, France).

### Ultrasound

The same US machine and probe were used in each center (Envisor^® ^point-of-care system with a 12 to 3 MHz linear array probe; Philips Medical Systems, Best, The Netherlands). The protocol required three operators: one dealt with the airway while the other two performed the US-guided PCT (an assistant and an operator). All physicians had the same level of experience of PCT (with over 50 operations) and the same university-level training in the use of US in anesthesia and critical care medicine.

### Complications

Patient care began with PCT and ended with decannulation. Complications were defined as minor, intermediate, or major and were further classified into technical, intra-procedural, or post-procedural incidents. Minor complications were defined as clinically irrelevant and clearly did not harm the patient. Intermediate complications were defined as potentially harmful for the patient. Major complications required medical or surgical intervention. The complications are listed and classified in Table 1.

**Table 1:** Classification of complications

Minor	Intermediate	Major
Bleeding not requiring compression or administration of packed red blood cells	Bleeding requiring compression without blood transfusion	Bleeding requiring administration of packed blood cells
Hypoxemia (SpO_2 _of less than 90%) or hypotension (systolic arterial pressure of below 100 mm Hg) for less than 5 minutes or both	Posterior tracheal wall injury but not requiring surgical repair	Esophageal injury
Difficult puncture or multiple punctures (more than three)	Subglottic stenosis	Posterior tracheal wall injury requiring surgical repair
Puncture of the tracheal tube cuff	Granuloma	Pneumothorax
Peristomal infection not requiring antibiotic treatment		Peristomal infection requiring local care or antibiotic treatment or both
Atelectasis	Malposition of the tracheostomy tube (pre-tracheal or para-tracheal insertion)	Loss of airway
		Surgical conversion
Tracheal ring fracture		Cardiac arrest
		Death

SpO_2_, oxygen saturation as measured by pulse oximetry.

### Study design

**Figure 1:**
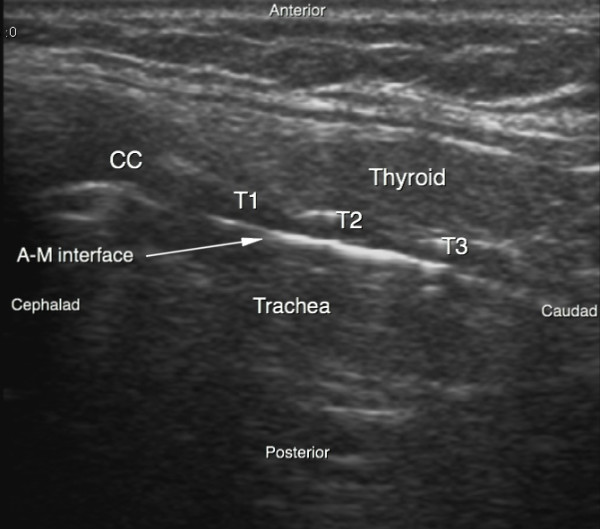
**Ultrasound sagittal view of the neck**. A-M interface, air-mucosa interface; CC, cricoid cartilage; T1, first tracheal ring; T2, second tracheal ring; T3, third tracheal ring.

**Figure 2:**
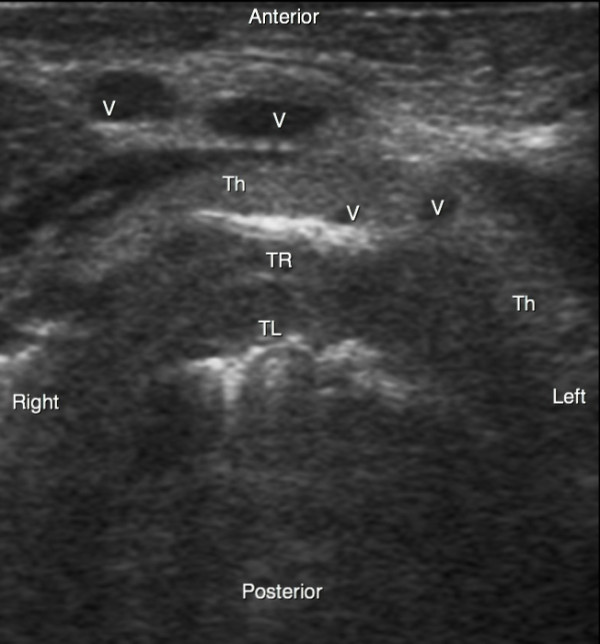
**Ultrasound transversal view of the neck**. Th, thyroid gland; TL, trachea lumen; TR, tracheal ring; V, vessel.

**Figure 3:**
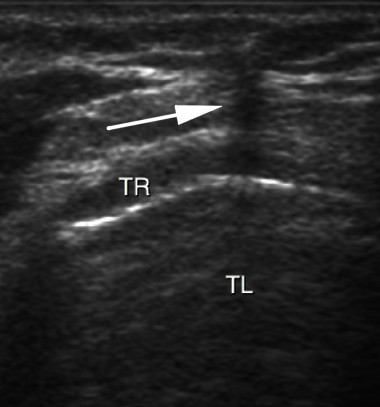
**Real-time ultrasound guidance using an out-of-plane approach**. Progression of the needle is determined by a distinct acoustic shadow (arrow). TL, trachea lumen; TR, tracheal ring.

**Figure 4:**
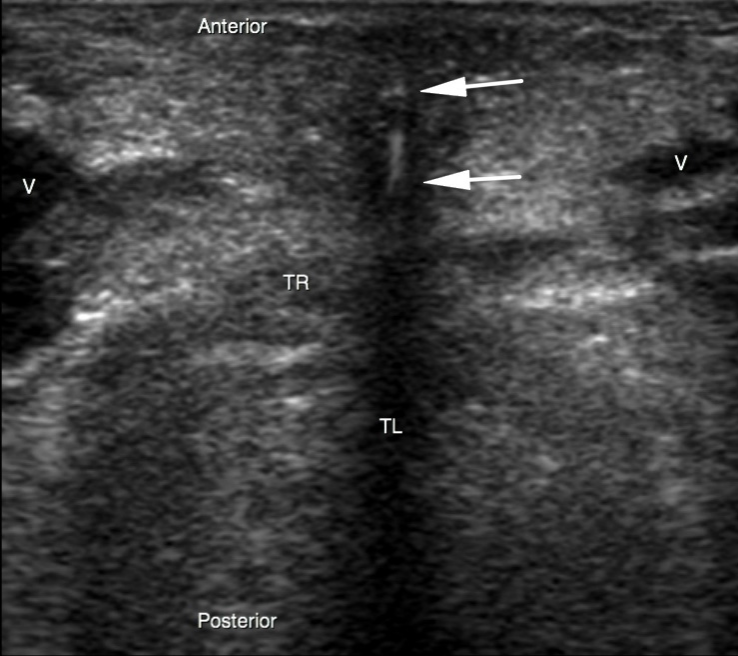
**Real-time ultrasound guidance using an out-of-plane approach**. The dilator is determined by a hyperechoic signal centered by a distinct acoustic shadow (arrows). TL, trachea lumen; TR, tracheal ring; V, vessel.

PCT was performed after deep sedation and analgesia by continuous infusion of propofol and sufentanil intravenous bolus (0.3 gamma/kg). Muscle relaxation was achieved with an intravenous bolus of cisatracurium (0.3 mg/kg). Patients were ventilated under volume-targeted mechanical ventilation with a 100% fraction of inspired oxygen (FiO_2_), and ventilatory parameters (tidal volume, respiratory rate, and positive end-expiratory pressure) were kept constant. Continuous hemodynamic monitoring (five-lead electrocardiogram, blood pressure, heart rate, and pulse oxygen saturation) was performed. After skin disinfection, a physician determined the point of puncture by palpation of standard anatomical landmarks. Prior to PCT, he performed a US examination of the neck region with longitudinal sections to locate the cricoid cartilage, the tracheal rings, and the puncture site (Figure 1). Then he performed US transversal sections to identify arteries, veins, thyroid, trachea, and endotracheal tube and measure the thickness of the skin to the anterior tracheal wall (Figure 2). After having determined the puncture site and noted whether there was a difference between the two methods, the physician standing at the patient's head withdrew the endotracheal tube's balloon from near the vocal cords under direct laryngoscopic guidance. Next, a second operator performed the PCT by using the single-stage dilator technique with US guidance. A puncture needle with a saline-filled syringe was introduced perpendicularly to the skin and advanced until the needle was seen to pass the anterior trachea wall during an aspiration of air. Then the needle was angled caudally to prevent retrograde passage of the guide wire. The needle was visualized in an 'out-of-plane' mode (that is, the needle path was determined by the presence of a distinct acoustic shadow ahead of the needle) on a transversal section of the neck region (Figure 3). The guide wire was introduced, the needle was removed, and a small horizontal incision was made at the point of puncture. The guide wire was visualized as a hyperechoic signal on transversal and longitudinal sections (Figure 4). The small dilator was then used to create the initial stoma followed by the single-stage curve dilator over the guide wire. The tracheostomy tube fitted over an appropriate loading tube was passed through the stoma. US provides information on the correct positioning of the puncture site and the guide wire before the dilatation of the trachea and then placement of the tracheostomy tube (Figure 4). Complications during the PCT procedure were monitored. An ear, nose, and throat specialist performed an endoscopic check before decannulation of the patient (even in dead patients) or before ICU discharge for non-decannulated patients.

### Data collection

The following data were collected: gender, age in years, height in meters, weight in kilograms, body mass index (BMI), the Simplified Acute Physiology Score II (SAPS II), diagnosis on hospitalization, duration of mechanical ventilation prior to PCT (in days), indication for tracheostomy, anatomical palpation data (short neck, palpated goiter, deviation of the trachea, vessels, puncture site, and cricoid-manubrium distance in centimeters), US data (thyroid, tracheal deviation, aberrant vessels, puncture site, subcutaneous tissue thickness (in centimeters) defined by the distance between the skin and the anterior wall of the trachea measured perpendicularly to the skin at the puncture level, tracheal diameter in centimeters, and installation time in minutes), the duration of the tracheostomy defined by the time (in minutes) between the puncture of the trachea and the ventilation of the patient, difficulty in achieving US-guided PCT (rated on a simple numerical scale; 1: easy; 2: a few difficulties in identifying anatomical structures and in implementation; 3: moderate difficulties in identifying anatomical structures; 4: very difficult; and 5: impossible), hemodynamic data before and after the completion of PCT (blood pressure in millimeters of mercury and heart rate in beats per minute), and complications.

### Statistical analysis

Data were expressed as the median (25th to 75th percentiles) or number (percentage). Obesity was defined as a BMI of at least 30 kg/m^2^. We compared the group of obese patients with the group of non-obese patients. A non-parametric Mann-Whitney test was used for inter-group comparisons of continuous variables. Categorical variables were compared by using a chi-squared test (and a Yates correction if necessary) or a Fisher exact test. A *P *value of not more than 0.05 was considered statistically significant. All statistical analyses were performed by using SPSS Statistics 18 (IBM Corporation, Armonk, NY, USA).

## Results

Fifty patients were prospectively enrolled between March 2010 and August 2011. Twenty-six patients were obese - median BMI of 34 kg/m^2 ^(32 to 38) - and five of the latter were morbidly obese. The median ages were 64 years (50 to 74) in the obese group and 58 years (46 to 64) in the non-obese group (*P *= 0.62) (Table 2). Of the 50 patients, 23 (46%) had been hospitalized for a medical problem (respiratory failure in chronic obstructive pulmonary disease, stroke, seizures, acute heart failure, and so on) and 27 (54%) had undergone cardiac, vascular, or digestive surgery or severe trauma. The most frequent indication for tracheotomy was difficult weaning from predictable, prolonged mechanical ventilation (due to a neurological etiology in 39% of cases). Table 2 presents demographic data on the study population.

**Table 2:** Demographic data for the overall study population and in the obese and non-obese subgroups

	Global population (n = 50)	Obese (n = 26)	Non-obese (n = 24)	*P *value
Age, years	62 (46-72)	64 (50-74)	58 (46-64)	0.62
Male gender	36 (72%)	19 (70%)	17 (74%)	0.4
Body mass index, kg/m^2^	31 (25-35)	34 (32-38)	25 (24-28)	< 0.001
SAPS II	41 (31-57)	40 (25-47)	47 (36-58)	0.06
Diagnosis on admission				0.9
Cardiogenic shock	1 (2%)	1 (4%)	0	
Acute respiratory distress syndrome	15 (30%)	10 (38%)	5 (20%)	
COPD failure	4 (8%)	2 (8%)	2 (8%)	
Neurological problem	3 (6%)	2 (8%)	1 (4%)	
Cardiac surgery	14 (28%)	8 (30%)	6 (25%)	
Vascular surgery	2 (4%)	1 (4%)	1 (4%)	
Thoracic surgery	3 (6%)	0	3 (13%)	
Visceral surgery	5 (10%)	2 (8%)	3 (13%)	
Polytrauma	3 (6%)	0	3 (13%)	
MV before tracheostomy, days	22 (16-29)	21 (16-29)	22 (19-29)	0.24
Time to decannulation, days	23 (15-32)	26 (13-34)	21 (16-31)	0.66

Values are expressed as the median (25th-75th percentiles) or as number (percentage). COPD, chronic obstructive pulmonary disease; MV, mechanical ventilation; SAPS II, Simplified Acute Physiology Score II.

### Anatomical and ultrasound data

Twenty-six patients (52%) had a short neck, and the median cricoid-manubrium distance was 5.5 cm (4.5 to 7). The prevalence of a short neck was higher in the obese group than in the non-obese group (64% and 38%, respectively; *P *= 0.05). The cricoid-manubrium distance was lower in the obese group than in the non-obese group: 5 cm (4.2 to 6) and 6 cm (4.7 to 7), respectively; *P *= 0.05 (Table 3). For the overall study population, the median thickness of subcutaneous tissue at the puncture site was 1.01 cm (0.77 to 1.45). These parameters differed significantly in obese patients, who had a greater pre-tracheal soft-tissue thickness (*P *= 0.01). The two groups did not differ significantly in terms of the tracheal diameter (Table 3).

**Table 3:** Anatomical and ultrasound-guided percutaneous tracheostomy data in the overall study population and in the obese and non-obese subgroups

	Study population (n = 50)	Obese (n = 26)	Non-obese (n = 24)	*P *value
Short neck	26 (52%)	17 (64%)	9 (38%)	0.05
Tracheal deviation	9 (18%)	4 (15%)	5 (21%)	0.72
Cricoid-manubrium distance, cm	5.5 (4.5-7)	5 (4.2-6)	6 (4.7-7)	0.05
Subcutaneous tissue thickness, cm	1.01 (0.77-1.45)	1.29 (0.96-1.59)	0.97 (0.68-1.15)	0.01
Trachea diameter, cm	2.1 (1.9-2.2)	2.1 (1.9-2.2)	2 (2-2.2)	0.24
Ultrasound examination time, minutes	10 (5-12)	10 (5-12)	10 (7-13)	0.83
Percutaneous tracheostomy time, minutes	10 (5-12)	9 (5-10)	10 (8-14)	0.1
Total time, minutes	18 (15-25)	17 (15-23)	20 (16-26)	0.36
Numerical scale^a^				0.5
1: Easy	24 (48%)	12 (46%)	12 (50%)	
2: A few difficulties	16 (32%)	8 (31%)	8 (33%)	
3: Moderate difficulties	9 (18%)	6 (23%)	3 (13%)	
4: Very difficult	1 (2%)	0	1 (4%)	
5: Impossible	0	0	0	

Values are expressed as the median (25th-75th percentiles) or as number (percentage). ^a^Numerical scale of difficulty in achieving ultrasound-guided percutaneous tracheostomy.

### The ultrasound-guided technique

The median total time was 18 minutes (15 to 25). The overall PCT procedure consisted of an initial US examination of the neck region, lasting 10 minutes (5 to 12), and a US-guided implementation phase, also lasting 10 minutes (5 to 12). These times were similar in the two groups (Table 3). The physician decided to change the puncture site in 25 cases (50%) because of tracheal coverage by the thyroid (n = 8, 32%) or aberrant vessels (n = 16, 64%) or tracheal deviation (n = 11, 44%) or a combination of these. Identification of the anatomical structures and the PCT guidance were considered to be easy in 24 patients (48%), moderately difficult in 16 patients (32%), difficult in nine patients (18%), and very difficult in one patient (2%). No difference was observed when comparing obese and non-obese patients. It was possible to carry out all PCTs with US guidance. No surgical conversions occurred.

### Complications

In terms of technical complications, we observed two multiple punctures (4%) and six punctures of the tracheal tube cuff (12%). Intra-procedural complications included desaturation for less than 5 minutes in three patients (6%), three cases of minor (< 5 mL) bleeding (6%), and three episodes of hypotension for less than 5 minutes (6%). None of the patients had severe bleeding. Post-procedural complications included a hematoma at the puncture site, followed by a skin infection (2%) that resolved easily with local care and intravenous antibiotics. Bronchoscopic check revealed one case (2%) of fracture of the cricoid without tracheal stenosis, a fractured tracheal ring (2%), and two cases of granuloma (4%). The obese and non-obese groups did not differ significantly in terms of these morbidity parameters (Table 4). The median times to decannulation were 26 days (13 to 34) in obese patients and 21 days (16 to 31) in non-obese patients (*P *= 0.66). Nine patients died from their disease. Nine patients (18%) were not decannulated during the study period.

**Table 4:** Complications in the overall study population and in the obese and non-obese subgroups

	Global population (n = 50)	Obese (n = 26)	Non-obese (n = 24)	*P *value
No complications	33 (66%)	17 (65%)	16 (71%)	0.92
Minor complications				
Hypotension	3 (6%)	1 (4%)	2 (8%)	0.6
Desaturation: SpO_2 _< 90%	3 (6%)	1 (4%)	2 (8%)	0.6
Tracheal cuff puncture	6 (12%)	2 (8%)	4 (16%)	0.41
Multiple puncture	2 (4%)	0	2 (8%)	0.22
Bleeding less than 5 mL	3 (6%)	1(4%)	2 (8%)	0.6
Fractured tracheal ring	2 (4%)	1 (4%)	1 (4%)	1
Atelectasis	1 (2%)	1 (4%)	0	1
Intermediate complications				
Granuloma	2 (4%)	2 (8%)	0	0.5
Major complications				
Cutaneous infection	1 (2%)	0	1 (4%)	1
Surgical conversion	0	0	0	
Death	0	0	0	
No complications	17 (34%)	9 (35%)	8 (33%)	0.92

Values are expressed as the number (percentage). SpO_2_, oxygen saturation as measured by pulse oximetry.

## Discussion

Our results demonstrate that PCT can be performed in obese patients under real-time US guidance and with a short completion time. The majority of complications were minor ones (hypotension, desaturation, minor bleeding, and puncture of the tracheal tube cuff) and did not differ in their prevalences in obese and non-obese patients. We did not observe any life-threatening complications.

Several studies have emphasized the value of pre-PCT US examination of the neck region to reduce the incidence of complications [14-16,20-25]. Recently, Rajajee and colleagues [18] demonstrated the feasibility of US guidance during the implementation of PCT in a population of neuro-intensive care patients. Our results confirm the feasibility of this procedure in a larger and more heterogeneous cohort of intensive care patients (even obese ones). Obesity is usually considered to be a relative contraindication to PCT because anatomical conditions can make it difficult to properly identify the patient's anatomical landmarks. Despite a shorter cricoid-manubrium distance, a greater trachea-skin distance, and a shorter neck in our study cohort, US was able to visualize the puncture level, the path of the needle, the tracheal puncture, and all other stages of the procedure (insertion of the needle, the dilator, and then the tube) in real time. Furthermore, the physicians reported that US was easy to use in 80% of cases, even in obese patients. The completion time for US-guided PCT was similar to that for PCT in the absence of US guidance [4]. Furthermore, the fact that the completion time in obese patients was similar to that recorded in non-obese patients emphasizes the feasibility of US guidance in this specific population.

By using standardized definitions to evaluate the incidence and severity of complications (particularly in obese patients), we observed a lower incidence of complications than reported in the literature [10,11]. In our study, the majority of complications were minor ones with little or no clinical significance (minor bleeding and short periods of hypotension or desaturation). None of the obese patients had major complications. Moreover, we did not observe late complications, such as tracheal stenosis. The use of US guidance may account for these results since this modality is probably more informative than bronchoscopy in terms of identification of the cervical anatomy and puncture site. Many surgical teams perform bronchoscopy-guided PCT. However, Walz and Schmidt [26] highlighted the discrepancy between the palpated landmarks and the actual level of puncture during fiber-optic monitoring. Massick and colleagues [27] found that the occurrence of complications was linked to the difficulty in identifying anatomical features. Given that obese patients have shorter and thicker necks, bronchoscopy transillumination cannot indicate the puncture site or identify pre-tracheal vessels. In a study by Byhahn and colleagues [10], bronchoscopy-guided PCT was five times more likely to result in serious adverse events in obese patients than in non-obese patients. Nevertheless, most of the complications could be anticipated by using US examination of the neck to identify the pre-tracheal tissue mass, subcutaneous vessels, and the trachea's axis. US has the advantage of identifying aberrant vessels (damage to which can otherwise cause bleeding [25,28]) and better defining the location and vascularization of the thyroid. In our cohort, US guidance prompted a change in the puncture site in 50% of patients in order to avoid pre-tracheal vessels and better center the PCT site. Romero and colleagues [29] used similar reasoning to demonstrate the safety of fiber-optic bronchoscopy-assisted PCT. However, in the latter protocol, patients underwent a pre-PCT US examination to identify vascular structures and select the optimal PCT site. The ability of US to provide this anatomical information may explain the low incidence of serious complications (such as major bleeding) observed in our obese patients. As with bronchoscopy, US allowed us to focus the puncture point on the trachea and thus avoid poor tracheal tube positioning. Hence, we did not observe any complications related to malposition of the tracheostomy (for example, pre-tracheal or para-tracheal insertion) or puncture of the posterior tracheal wall.

One limitation of the use of US is the required level of anatomical knowledge of the neck (especially among critically ill obese patients). All of the practitioners in our study had been trained by a radiologist on the anatomy of neck as revealed by US. Even so, the cervical anatomy was identified with great difficulty in 10 patients (20%) but did not prevent PCT. There were several reasons for this great difficulty. Short, thick necks limited our ability to carry out a US examination. The presence of a large, hypoechoic, anterior venous maze could have hindered guidance of the needle. Another difficulty was in identifying the balloon of the endotracheal tube with sonography; the tracheal tube cuff was perforated in six cases (12%). Visualization of the balloon was partly improved by using a double-contrast technique (that is, a balloon inflated with water). However, this technique was non-optimal because the air around the tracheal tube could act as an artefact for US. Two other studies reported a rate of tracheal tube cuff puncture of 13% to 17% for bronchoscopy-guided PCT [30,31]. Even though US cannot correctly identify the tracheal tube cuff, the use of this modality did not seem to increase the rate of tracheal tube cuff puncture. One patient suffered a cricoid fracture; the chosen puncture site was moved nearer to the head because of the thickness of the US probe and the guiding in out-of-plane mode. The lack of differences between obese and non-obese patient groups in terms of the completion time, difficulty rating, and incidence of complications prompts us to consider that obesity is not a limiting factor for the use of US guidance. Our study included few morbidly obese patients who may represent a group of patients for whom US would be more informative. None of the five morbidly obese patients had complications. Another limitation of our prospective cohort study related to the fact that we did not use a high-powered, non-inferiority methodology with an adequate sample size. However, our goals were to evaluate the feasibility of US guidance and to describe the implementation difficulty and occurrence of complications in a critically obese population rather than demonstrate the non-inferiority of US guidance relative to fiber-optic bronchoscopy guidance. Furthermore, at the time we designed our study, there were no available data on US-guided PCT in this type of population. The indications and contraindications are yet to be determined in larger cohorts. The role of US guidance (particularly in relation to bronchoscopy) remains to be clarified in prospective studies. The extrapolation of our results to other teams (with little or no training in US or cervical anatomy) might be a concern. A learning curve might well be required before the technique can be incorporated into routine use.

## Conclusions

US-guided PCT can be performed in obese patients, and the incidence of complications is low. Use of US provides a better understanding of the anatomy of the neck, prevents vascular puncture, and helps guide the tracheostomy procedure. Obesity does not appear to limit the implementation of US-guided tracheostomy.

## Key messages

• Ultrasound-guided percutaneous tracheostomy is feasible in obese patients and has a low complication rate.

• Ultrasound provides a better understanding of the anatomy of the neck, prevents vascular puncture, and helps guide the tracheostomy procedure.

## Abbreviations

BMI: body mass index; ICU: intensive care unit; PCT: percutaneous tracheostomy; US: ultrasound.

## Competing interests

The authors declare that they have no competing interests.

## Authors' contributions

P-GG conceived of the study, participated in its design and coordination, and drafted the manuscript. EZ, SP, J-PM, A-MG, RZ, and PM participated in the coordination of the study and in data acquisition and helped to draft the manuscript. HD participated in the study design, data collection, statistical analysis, and manuscript revision. All authors read and approved the final manuscript.
